# Metamizole-induced agranulocytosis from a German intensive care perspective

**DOI:** 10.3389/fmed.2026.1765803

**Published:** 2026-04-01

**Authors:** Laura Hille, Martin J. Hug, Tobias Wengenmayer, Hanna Hilger, Paul Marc Biever

**Affiliations:** 1 Interdisciplinary Medical Intensive Care, Medical Center - University of Freiburg, Faculty of Medicine, University of Freiburg, Freiburg, Germany; 2 Pharmacy, Medical Center - University of Freiburg, Freiburg, Germany

**Keywords:** agranulocytosis, dipyrone, Germany, intensive care unit, metamizole sodium, prescription statistics

## Abstract

**Background/objectives:**

Metamizole (dipyrone) is a non-opioid analgesic and antipyretic widely used in Germany, with prescriptions having increased more than tenfold since 1997. Despite its popularity, metamizole remains controversial due to the risk of agranulocytosis–a potentially life-threatening adverse reaction that can occur independently of dose or duration of therapy. This study aimed to describe severe cases of metamizole-induced agranulocytosis requiring intensive care treatment and to contextualize them within current consumption data.

**Methods:**

A retrospective analysis was conducted of all patients admitted to the interdisciplinary medical intensive care units of a tertiary university hospital in southern Germany between April 2022 and April 2025 due to metamizole-associated agranulocytosis. Demographic, clinical, and laboratory data were collected. Bone marrow examinations, microbiological findings, treatment regimens, and outcomes were analyzed descriptively.

**Results:**

Seven patients required intensive care for metamizole-induced agranulocytosis. All presented with profound neutropenia and severe infections. Two patients died from multiorgan failure despite maximal supportive care. The five survivors experienced prolonged ICU stays (11–60 days) with complications including septic shock, acute respiratory distress syndrome, and renal failure requiring dialysis. None of the patients had pre-existing hematologic disorders or immunosuppression.

**Conclusion:**

Metamizole-induced agranulocytosis is a rare but potentially severe adverse reaction. Even though its absolute incidence is low relative to the widespread use of metamizole, affected patients may experience life-threatening complications. Awareness of this risk, combined with careful benefit–risk assessment and consideration of alternative analgesics, remains essential for safe and rational use of metamizole in clinical practice.

## Introduction

**FIGURE 1:**
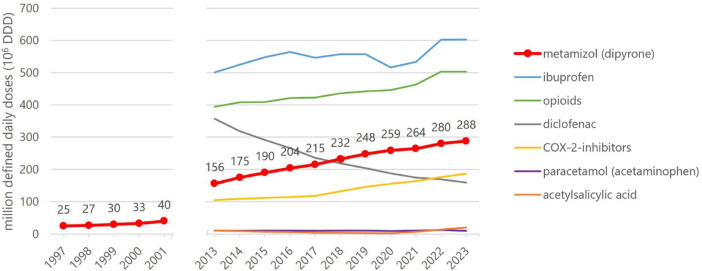
Prescriptions of analgesics and anti-inflammatory drugs in Germany [modified from (1)]. Shown are prescription-only drugs reimbursable under statutory health insurance.

Metamizole (dipyrone) was introduced to the market in 1922 by Hoechst under the trade name Novalgin^®^. It is a widely used analgesic and antipyretic, with prescription rates in Germany having increased significantly in recent years. From 25 million defined daily doses (DDD) in 1997, consumption rose almost linearly to 288 million DDD in 2023 – more than a tenfold increase (Figure 1) (1). Despite its popularity, the safety of metamizole remains controversial due to potentially severe adverse drug reactions, particularly agranulocytosis. Countries such as the USA, Canada, Australia, Japan, and some EU nations have withdrawn metamizole from the market due to these safety concerns.

In 2024, the European Medicines Agency (EMA) initiated a risk assessment procedure to review the benefit-risk profile of metamizole across the EU and possibly issue harmonized recommendations (2). Pharmacovigilance data indicate that agranulocytosis under metamizole therapy can occur regardless of dose or treatment duration, making estimation of its prevalence challenging (3). Reported incidence rates vary widely – from approximately 1 case per 1,500 treatments (4, 5) to 1 case per 1 million (6) treatments.

The pathophysiology of metamizole-induced agranulocytosis is not fully understood. Current evidence suggests an immune-mediated mechanism, potentially involving drug-dependent antibodies directed against neutrophils or their precursors (7). Additional hypotheses include direct toxicity of reactive metamizole metabolites on granulopoiesis (8) and a genetic susceptibility predisposing certain individuals to this adverse reaction (9). Bone marrow findings typically demonstrate selective aplasia or severe suppression of the granulocytic lineage, with preserved erythropoiesis and megakaryopoiesis (10).

This article presents a series of severe, intensive care unit-requiring cases of metamizole-induced agranulocytosis and places them in the context of current consumption data.

## Methods

A retrospective analysis was performed of all patients admitted to our interdisciplinary internal medicine intensive care units (31 beds) between April 2022 and April 2025 due to metamizole-associated agranulocytosis. The intensive care units provide tertiary care at a university hospital, serving as a regional referral center for an estimated catchment population of about one million inhabitants in southern Germany. Demographic data, indication, clinical course, and outcomes were collected.

Cases were identified retrospectively using the institutional electronic patient information system (MeDoc), which allows full-text searches of all discharge summaries. A structured keyword search using the German terms for metamizole and agranulocytosis (“*Metamizol*” and “*Agranulozytose*”) was performed. All retrieved discharge summaries were reviewed manually by the authors.

Only cases in which metamizole-induced agranulocytosis was documented as the final clinical diagnosis were included. Metamizole-induced agranulocytosis was defined as severe neutropenia or agranulocytosis occurring after exposure to metamizole, with a clear temporal association.

Cases were excluded if alternative causes of agranulocytosis were considered plausible, including hematologic malignancies, chemotherapy, or other potentially myelotoxic drugs.

Where available, bone marrow aspiration demonstrating aplastic or markedly suppressed granulopoiesis was considered supportive of the diagnosis. In all cases, the diagnosis was established in interdisciplinary consultation with a hematologist.

Given the descriptive nature of this case series, no formal hypothesis testing was performed. Continuous variables are reported as medians with interquartile ranges or mean with standard deviation.

Ethical approval was not required for this study in accordance with the local legislation and institutional requirements. The case descriptions reflect routine clinical practice.

## Results

Seven patients were admitted to intensive care for metamizole-associated agranulocytosis during the observation period.

None of the affected patients had relevant hematologic or immunologic pre-existing conditions, and none were receiving immunosuppressive therapy at the time of metamizole exposure. Most patients had few or no significant comorbidities.

Bone marrow aspiration was performed in six of the seven patients, each showing aplastic granulopoiesis, which was deemed consistent with metamizole-associated agranulocytosis by the evaluating pathologists. There was no evidence of alternative differential diagnoses such as hematologic malignancies, autoimmune disorders, or infectious causes. All patients received G-CSF (granulocyte colony-stimulating factor) to stimulate granulopoiesis.

All patients experienced severe infections due to the resulting immunosuppression, leading to complex and prolonged intensive care treatments. Additional patient characteristics are shown in Table 1.

**TABLE 1:** summarizes additional key characteristics.

Patient number	1	2	3	4	5	6	7	Overall
Age	80	52	24	44	74	62	62	56.9 (±18.9)^1^
Sex	Female	Female	Male	Female	Female	Male	Male	
Pre-existing conditions	Arterial hypertension, gonarthrosis	None	None	Obesity	COPD, rheumatoid arthritis, osteoporosis, dementia	Arterial hypertension, gonarthrosis	None	
Period from first metamizole intake to ICU admission [days]	20	52	23	4	6	42	12	20 (6–42)^2^
Indication for metamizole therapy	Knee replacement (knee TEP)	Post-zoster neuralgia	Odontectomy (wisdom tooth extraction)	Sore throat	High fever after insect bite	Knee replacement (knee TEP)	Rotator cuff rupture	
Leukocyte count at admission [10^3^/μl]	0.25	0.13	0.35	0.33	0.5	0.18	0.32	0.29 (±0.12)^1^
Time until normalization of leukocyte count [days]	11	10	12	15	10	8	5	10 (8–12)^2^
SOFA score at admission	6	10	10	14	14	8	19	11.6 (±4.4)^1^
Vasopressor agent	NorEpi, Vaso	NorEpi, Vaso	NorEpi	NorEpi, Vaso, Epi	NorEpi	NorEpi, Vaso, Epi	NorEpi	
Vasopressor duration [days]	9	12	3	30	18	22	17	17 (9–22)^2^
MV	Invasive MV	Invasive MV	None	Invasive MV	Invasive MV	Invasive MV	Invasive MV	
MV duration [days]	10	7	–	51	5	23	16	13 (7–23)^2^
Other extracorporeal organ support	None	Dialysis	None	VVA-ECMO; dialysis	None	VA-ECMO, Impella, dialysis	Dialysis	
ICU length of stay [days]	12	11	25	60	22	40	23	23 (12–40)^2^
Status at ICU discharge	Deceased (exitus letalis)	Deceased (exitus letalis)	Discharged at home	Discharged to rehabilitiation center	Discharged to rehabilitiation center	Discharged to general ward	Discharged to general ward	

Key patient characteristics. Values are presented as mean (±SD)^1^ or median (IQR)^2^. COPD, chronic obstructive pulmonary disease; Epi, epinephrine; ICU, intensive care unit; MV, mechanical ventilation; NorEpi, norepinephrine; TEP, total endoprosthesis; Vaso, vasopressin; V(V)A-ECMO, veno-(veno-)arterial extracorporeal membrane oxygenation.

- Case 1: An 80-year-old female developed bacterial sepsis with *Staphylococcus aureus* and *Klebsiella pneumoniae*, accompanied by impaired consciousness and delirium. She died following hypoxia-induced cardiac arrest after aspiration.
- Case 2: A 52-year-old female died from fulminant multiorgan failure (MOF) with acute kidney injury (AKI), *Escherichia coli* bacteremia, candidemia, acute respiratory distress syndrome (ARDS), and liver failure.
- Case 3: A 24-year-old male student developed severe sepsis following a peritonsillar abscess and additionally suffered a severe soft tissue abscess and *E. coli* bacteremia.
- Case 4: A 44-year-old female with moderate obesity developed severe ARDS under *Pseudomonas aeruginosa* infection, requiring VV-ECMO therapy, and experienced septic shock with subsequent MOF, dialysis-dependent renal failure, septic cardiomyopathy, and microangiopathic necrosis of both forefeet–resulting in toe amputations.
- Case 5: A 74-year-old female with relevant comorbidities developed septic shock due to pneumonia requiring invasive ventilation, as well as *Enterococcus faecium* bacteremia.
- Case 6: A 62-year-old male survived a 42-days ICU stay after severe septic shock due to Fournier’s gangrene, septic cardiomyopathy requiring VA-ECMO and Impella support, dialysis, rectal ischemia, and invasive aspergillosis.
- Case 7: A previously healthy 62-year-old male suffered severe septic shock from pneumonia with *Serratia marcescens*, complicated by in-hospital cardiac arrest during repositioning, dialysis-dependent renal failure, and acute liver failure.

## Discussion

This case series highlights the potentially life-threatening risks of metamizole therapy. All seven patients developed severe infectious complications as a consequence of profound agranulocytosis. However, no uniform clinical course, characteristic complications, or pathogen spectrum specific to metamizole-induced agranulocytosis was observed, with infections largely reflecting opportunistic and nosocomial exposures typical for critically ill neutropenic patients. Two patients died despite maximal intensive care management. The five survivors all had prolonged and complex ICU courses associated with significant complications, suggesting likely long-term impairments in physical and cognitive function. Clinical stabilization and discharge from intensive care were closely linked to recovery of bone marrow function, with resolution of agranulocytosis representing the key prerequisite for improvement.

Interestingly, six of seven cases in this series were referrals from external hospitals for the treatment of metamizole-induced agranulocytosis. During the observation period, no cases of agranulocytosis occurred among patients treated with metamizole in our own ICU, despite its frequent use.

These severe courses must be interpreted in the context of the steadily increasing use of metamizole in clinical practice, as illustrated by national prescription data (Figure 1). With rising exposure, awareness of rare but potentially life-threatening adverse drug reactions becomes increasingly relevant, particularly in intensive care settings.

While the indication for analgesia or fever management was reasonable in all cases described, alternative agents with a more favorable safety profile might have been considered.

The choice of analgesic/antipyretic must always be individualized, taking into account patient-specific factors such as pain intensity, hepatic and renal function, co-medications, cardiovascular comorbidities, allergies, etc. Established alternatives including paracetamol, NSAIDs, and opioids remain important components of multimodal pain management. Nevertheless, metamizole remains a valuable option in the intensive care setting, where severe pain, fever, and spasm-related symptoms are common and contraindications to alternative agents frequently limit therapeutic choices.

Reported estimates of the frequency of metamizole-induced agranulocytosis vary widely across different study designs and populations, as recently confirmed by the EMA (11). In a large prospective surveillance study from Germany, an incidence of approximately 1 case per 1,000,000 person-years was observed (6), while analysis of European pharmacovigilance data has suggested reporting rates in the range of approximately 1 per 10,000 to 1 per 100,000 exposed patients depending on reporting patterns (3). At the other end of the spectrum, prescription-based estimates from Sweden and Germany have suggested substantially higher risks, on the order of approximately 1 case per 1,400–1,600 prescriptions – findings that contributed to regulatory actions in some countries (4, 5). This heterogeneity likely reflects differences in study methodology, case definitions, denominators (e.g., prescriptions, treated patients, or person-years), as well as underreporting, country-specific prescribing patterns, and variation in exposure and patient populations (12, 13). Taken together, these factors make it difficult to derive a precise and universally applicable estimate of the true incidence of metamizole-induced agranulocytosis.

This study has several limitations. Its retrospective, single-center design and the small number of cases limit generalizability. As a descriptive ICU-based case series, it does not include a control group and cannot provide incidence or risk estimates, as reliable denominator data on metamizole exposure were not available. Case identification relied on discharge summaries and manual review, so underreporting cannot be excluded. Finally, all patients were referred from external hospitals to a tertiary care referral center, introducing referral bias and potentially enriching for severe disease courses.

## Conclusion

Metamizole is a potent analgesic with additional antipyretic and spasmolytic properties and represents a valuable therapeutic option in the intensive care setting, where patients frequently suffer from severe pain and where contraindications to alternative analgesics are common. However, metamizole-induced agranulocytosis is a rare but potentially life-threatening adverse drug reaction. In critically ill patients, early recognition is particularly important, as clinical manifestations may overlap with other common ICU-related conditions such as sepsis or infection-related leukopenia. Metamizole should therefore be used as an option rather than adopted uncritically as standard therapy, with heightened clinical awareness and prompt diagnostic evaluation to reduce morbidity and improve outcomes.

## Data Availability

The raw data supporting the conclusions of this article will be made available by the authors, without undue reservation.
